# Preparing undergraduate students for clinical work in a complex environment: evaluation of an e-learning module on physiotherapy in the intensive care unit

**DOI:** 10.1186/s12909-020-02035-2

**Published:** 2020-04-28

**Authors:** Mel E. Major, Stephan P. J. Ramaekers, Raoul H. H. Engelbert, Marike Van der Schaaf

**Affiliations:** 1 European School of Physiotherapy, Faculty of Health, Amsterdam University of Applied Sciences, Amsterdam, the Netherlands; 2 Center of Expertise Urban Vitality, Faculty of Health, Amsterdam University of Applied Sciences, Amsterdam, the Netherlands; 3 Department of Rehabilitation, Amsterdam Movement Sciences, Amsterdam University Medical Centers, Amsterdam, the Netherlands

**Keywords:** E-learning, Intensive care unit, Undergraduate education, Physiotherapy, Competencies

## Abstract

**Background:**

Intensive Care Units (ICUs) are daunting environments for physiotherapy (PT) students performing clinical rotations. To prepare students for this environment, a newly developed, evidence-based e-learning module was designed and implemented in the undergraduate curriculum. The aim of this study was to investigate whether e-learning is a feasible method in preparing PT students for clinical work in complex ICU environments, as perceived by students and experts.

**Methods:**

A mixed methods proof of concept study was undertaken. Participants were final-year students of an international curriculum, and experts from didactic and clinical fields. An e-learning module consisting of 7 separate chapters based on the latest scientific evidence and clinical expertise was developed, piloted and incorporated into the undergraduate curriculum as a compulsory course to be completed prior to clinical ICU rotations. Data were collected through 3 focus group meetings and 5 semi-structured interviews; these meetings and interviews were audio recorded, transcribed verbatim and analyzed.

**Results:**

The study sample comprised of 14 students and 5 experts. Thematic analysis revealed three themes: *expected competencies of PT students in ICU*, *feeling prepared for ICU clinical work* and *dealing with local variety.* The e-learning module enabled students to anticipate clinical situations and PT tasks in the ICU. Higher level clinical reasoning skills, handling of lines and wires and dealing with out-of-textbook situations could not be achieved with the e-learning module alone.

**Conclusions:**

An e-learning module can sufficiently prepare PT students for their clinical tasks in the ICU, as long as it is integrated with, or closely connected to, the students’ clinical placement.

## Background

Developments in intensive care medicine over the past decades have led to both an increase in complexity as well as in the number of patients surviving critical illness, resulting in prolonged intensive care unit (ICU) stays [1, 2]. Evidence on the effects of prolonged immobility in these vulnerable patients is abundant [2–6] and multidisciplinary interventions directed to early mobilization of ICU patients are globally implemented [7–10]. Physiotherapists, as part of a rehabilitation team, are essential to ICUs.

Recent studies have identified physiotherapy (PT) competencies required for the ICU setting and recommendations have been made to define a professional profile specifically for ICU physiotherapists (ICU-PTs) [1, 11–15]. Across these studies, consensus exists on the necessity for ICU-PTs to have knowledge and understanding of medication interaction, pathophysiology, ICU equipment (including mechanical ventilation modes), laboratory testing and imaging investigations. ICU-PTs should also be familiar with practicing PT within safety parameters and based on sound clinical reasoning [12–16].

Undergraduate PT curricula need to be adaptive to constantly changing clinical environments and requirements, but also include authentic learning experiences to optimally prepare graduates for the profession [17, 18]. Teaching methods currently applied in preparation for the ICU include theoretical classes, classroom and in-hospital simulations with computerized manikins and compulsory clinical rotations [19–22]. Despite having had preparational classes or simulation scenarios, PT students are often overwhelmed by the ICU environment and lack confidence in the execution of clinical tasks involving (sedated) patients dependent on mechanical ventilation [23, 24].

E-learning modules use a variety of interactive teaching methods, such as real-life videos, which can be helpful in transferring knowledge and reasoning skills and preparing students for complex environments. It has been shown to be an effective and flexible teaching tool in undergraduate medical and allied health education [25–29]. With internet-based courses being accessible anywhere in the world and on any electronic device [30, 31], an e-learning module on ICU-PT could be a convenient teaching method for an internationally oriented curriculum. Therefore, the aim of this study was to develop, implement and evaluate an e-learning module on evidence-based physiotherapy in the ICU and to investigate its feasibility with regards to preparing undergraduate PT students for clinical work in ICUs worldwide.

## Methods

**Fig. 1:**
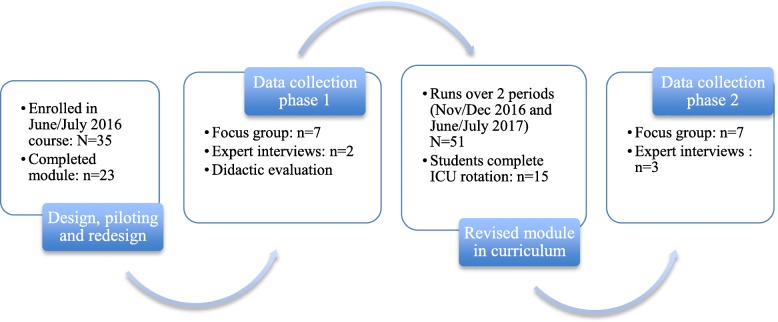
Phases of study: design and evaluation

A mixed method proof of concept study was undertaken in the period of June 2016 until January 2018. Figure 1 shows an overview of the different study phases.

### Context

In 2016, the undergraduate curriculum of the European School of Physiotherapy (ESP), Amsterdam University of Applied Sciences (AUAS), did not contain a course specific to PT responsibilities in the ICU. Relevant content, such as cardiorespiratory physiotherapy and pathophysiology, were embedded within different parts of the curriculum, instead of focused into one course module. Undergraduate students in the ESP program are required to conduct 4 clinical rotations, each lasting 10 weeks. The last two clinical rotations are commonly conducted within rehabilitation facilities, a hospital stroke unit and/or an ICU. As these rotations can take place anywhere in the world, students are provided with internationally oriented course content. Evaluation results led to the decision to design an e-learning module, ‘Physiotherapy in the ICU’, to be completed before students’ practical ICU placements.

### Design of the e-learning module

Clinical, didactic and research experts provided input on the e-learning module. Core topics, competencies and learning objectives, according to Bloom’s revised taxonomy [32], were identified (Table 1). Content decisions were based on recent medical and allied health research evidence for physiotherapy in the ICU and aimed to provide a general introduction into the topic. Videos were recorded at the ICUs at the Amsterdam University Medical Centers (Amsterdam UMC) and informed consent for recording was obtained from patients and clinicians.

**Table 1:** Learning objectives e-learning module ‘Physiotherapy in the ICU’

Objectives	Bloom’s revised taxonomy
1) Has insight in safety criteria in the ICU setting and can determine and justify a GO or NO GO for physiotherapy	Remembering, Understanding, Applying
2) Has understanding of the place and content of physiotherapeutic screening and assessment within the ICU	Understanding
3) Has insight in evidence-based physiotherapeutic interventions in the ICU for both conscious and unconscious patients	Understanding
4) Applies clinical reasoning skills within the complex environment of the ICU patient	Understanding, Applying
5) Understands the importance of multidisciplinary collaboration with regards to the ICU patient	Understanding

### Key features of the e-learning module

The final module contained 7 chapters: *general introduction to ICU*, *the impact of ICU admission on patient and family*, *introduction to ICU rehabilitation*, *physiotherapeutic assessment*, *physiotherapeutic interventions*, *the post-surgical patient* and *reporting and interprofessional collaboration*. A variety of teaching materials was incorporated in the e-learning module to facilitate student learning and motivation: interactive assignments, background literature and short quizzes, self-developed and online videos (patient-testimonials, patient and PT observations, skills-modelling videos), and text presented in presentation slide format, narrated by a native English speaker. Each module finished with a quiz and direct feedback was provided. An online community supported the e-learning module, operating as a forum between lecturer and students where additional material, as well as content and technical questions, could be posted.

A 25 question, multiple choice online exam consisting of 3-answer options was developed as a means to test students on obtained knowledge and reasoning skills related to the learning objectives and predetermined exam matrix. Students required a passing mark before being allowed to start the clinical rotation. After the clinical rotation, students submitted a mandatory case report about one of the ICU patients that they had observed during their placement. The mark received for the case report determined the final ICU course grade.

### Technical details of the e-learning system

In 2015, a trial version of the e-learning module was developed in Articulate Storyline© (version 2015) by a trainee developer of the Institute Information Communication Technology and Education (ICTO) of the Amsterdam UMC. Microsoft PowerPoint presentations, supported by audio files, videos and interactive assignments, formed the content of this trial version. The module was accessed through Google Sites. ICU clinicians and a small sample of students (*n* = 8) provided feedback on content accuracy, user-friendliness and overall aesthetic of the module.

In 2017, the final version of the e-learning module was incorporated in G Suite from Google Cloud©, with additional interactive assignments created in Adobe Activate©. The main reason for the transition to G Suite was the flexibility of the system, enabling easy content updates as new evidence on physiotherapy practices in the ICU emerged.

### Data collection phase 1

The aim of this study phase was to evaluate the e-learning module on user-friendliness, level of complexity, didactic alignment and accessibility. Between June and October 2016, the e-learning module was piloted among a group of undergraduate students (*n* = 23, male: 5, female: 18) for whom the module was not a compulsory part of the BSc curriculum. Information was obtained through focus group (FG) sessions, semi-structured interviews and exam analysis, which led to technical changes to improve user-friendliness. The key features of the course remained unchanged.

### Data collection phase 2

The course was implemented in the curriculum of the academic year 2016–2017. It ran twice for a period of 6 weeks and was evaluated during data collection phase 2. Participants were recruited via e-mail; only students who had completed both the e-learning module and a clinical rotation between November 2016 and July 2017 were included. Clinical experts were purposefully sampled through the faculty’s international network. Students and experts were provided with information explaining the aim of the study. Participating experts received PDF files containing the e-learning module’s content in preparation for the interview.

Interview guides were set up for FG meetings and semi-structured interviews (Table 2). Interviews were conducted by a research assistant, either face-to-face or via Skype©, and had a duration of 30–60 min. FG sessions, lasting approximately one hour per session, took place at the Faculty of Health, AUAS, in Amsterdam. Sessions were moderated by one of the research assistants and monitored by the main investigator.

**Table 2:** Interview guide and topic list expert interviews and focus groups

Method	Topic	Example question
Interview	**Current role of the expert in ICU** • Tasks and responsibilities • Clinical practice hours **Expectations of PT students on clinical rotations** • Knowledge • Skills • Common mistakes **Evaluation of e-learning module’s content** • Agreement • Disagreement • Suggestions for improvement **Specifics to expert’s ICU context** • International differences / diversity • Skills/Knowledge	*Can you start by describing what your daily tasks and responsibilities within the ICU are?* *What knowledge do you feel a PT student should have when doing a rotation in ICU?* *How did you perceive the information presented in the ICU online course?* *Are there distinct skills a PT student should possess when doing a rotation in the ICU department at your hospital?*
Focus Group	**Evaluation of e-learning module’s content** • Positive/negative • Points for improvement **Experience of ICU rotation** • Supervision • Matching content e-learning module and real-life situation • Specific experience that stood out	*What did you like best about the e-learning module’s content? And what did you not like?* *How did you feel during the rotation in ICU?* *How much do you think the e-learning module prepared you for the ICU environment?*

### Data analysis

Interviews and FG sessions were audio- or video recorded and transcribed verbatim. Initial line-by-line coding was followed by focused coding in order to identify categories and transcendent themes from the data. Regular feedback sessions were scheduled with the complete research team to discuss the data analysis and to facilitate a thorough categorization, interpretation and establishment of data saturation. MAXQDA12 was used for qualitative data analysis. The online exam was analyzed for estimated internal consistency reliability (Cronbach’s Alpha) between questions, proportions of correct answers and standard measurement error. *P*-values (difficulty index) corrected for chance score (expressed as *Pc*), point-biserial correlations (item discrimination index) and distractor efficiency (DE), and were calculated for the exam. Individual exam scores were checked to reveal deviant response patterns. If indicated by the exam statistics, the content of a question was reviewed to establish its validity. No questions were removed from the exam.

### Ethics

Ethical approval has been obtained from the ethical review board of the Netherlands Association for Medical Education (NVMO-ERB) file number 728. Written, informed consent was obtained from each participant.

## Results

In total, 14 international bachelor students participated in three FG sessions during study phases 1 and 2. The mean age was 25.3 (SD ± 3.2) and 93% (*n* = 13) of the students were female. Compared to gender distribution in the ESP program (female 59%, male 41%, 2019–2020 data), the proportion of females in this study was high. This can be explained by the higher number of females volunteering to participate in the 2016 pilot (19 out of 23) and more female students having completed the ICU rotation at the time of recruitment. Therefore, more females met the eligibility criteria for participation. All participating students were in their 3rd or 4th year of study. Purposive sampling of didactic and clinical experts led to participation of 5 experts in total (60% female, mean age 37, SD ± 7.8) (Table 3).

**Table 3:** Participant characteristics

Phase	Pilot: FG	Pilot: EI	Implementation: FG	Implementation: EI
Total N	7	2	7	3
Gender	Female: 6 (86%)	Male: 2 (100%)	Female: 7 (100%)	Female: 3 (100%)
Nationality	Italy (1) Ireland (1) Netherlands (1) Romania (1) Lithuania (1) Germany (1) South Africa (1)	Germany (1) Israel (1)	UK (1) Singapore (1) Greece (1) Germany (2) Netherlands (1) France (1)	South Africa (1) Ireland (1) Greece (1)
Role	Bachelor student (7)	Lecturer (1) Web developer (1)	Bachelor student (7)	ICU physiotherapist and clinical tutor (3) PhD student (1) Postdoctoral research fellow (1)
Mean age	26 (SD ± 4.0)	32.5 (SD ± 7.7)	25.7 (SD ± 2.75)	40 (SD ± 7.5)
Years of clinical experience	n/a	n/a	n/a	Mean: 17 (SD ± 9.2)

*FG* focus group, *EI* expert interview

### Results data collection phase 1

Qualitative analysis of transcripts of data collected in phase 1 showed that the content of the e-learning module was perceived positively with regards to: accessibility, degree of difficulty, variation in study assignments, encouragement of further learning and triggering curiosity, and transparency of the module’s learning objectives.

Didactic alignment was assessed as the alignment between the course objectives and the content of the online exam. Analysis showed that the exam did not align well; only 11 out of 25 questions corresponded with the course objectives. Feedback included suggestions to add case-based exam questions, which was done for the revised version. The learning objectives and testing matrix were fine-tuned and after revision, the exam construct and the didactic alignment improved (18 out of 25 corresponded precisely with the learning objectives). Analysis showed high internal consistency (Cronbach’s alpha: 0.84) with a tendency towards questions being too easy (*Pc =* 0.86).

### Results data collection phase 2: thematic analysis

The following themes were identified: *expected competencies of PT students in ICU*, *feeling prepared for ICU clinical work* and *dealing with local variety*.

#### Expected competencies of PT students in ICU

Participants’ perceptions regarding competencies expected of PT students in the ICU revolved around understanding theory, clinical performance, communication and clinical reasoning. With regards to theoretical knowledge, participants mentioned the understanding of physiological processes, interpretation of patient data and understanding of the ICU environment and equipment were the most important:

*“So, I think the main thing that would be good [is] if students had a good grip on [..] physiology and the theoretical...[..] [because] it is a pain to teach them the theoretical underpinnings [in] here.” (Amy, Irish clinical expert)*

*“I expect them [..] to have a good knowledge of the assessment [..] so looking at all systems so they can adequately clinically reason what they need to do [..]” (Wendy, South African clinical expert)*

*“He must have knowledge of the environment [..] and the difference between the ICU and the wards ..uhm.. mechanical ventilation.” (Alexandra, Greek clinical expert)*

With regards to clinical performance, participants agreed that (understanding of) cardiopulmonary assessment and interventions were required competencies.

*“[my clinical site] heavily focuses also on respiratory management [..] So they were like ‘oh, you don’t know how to do auscultation as an assessment?’ [..] so there’s like a whole system of respiratory physio that I had no idea about … [Have] Auscultated maybe once in school...” (Student: Grace)*

Being able to connect with the (unconscious) patient and communicate with the patient, family member and ICU staff, was found to be an essential competency by both clinical experts and students.

*“Communication I guess with the patient, especially if they’ve altered consciousness [..]. But even so, even when English is their first language, they can use medical jargon when communicating with patients.” (Amy, Irish clinical expert)*

*“And they need to [..] be able to be effective communicators in terms of, cause that’s part of, part of working in the ICU[..]” (Wendy, South African clinical expert)*

*“..talking to them [the patient] or finding a way to connect to them, ask them about their night or their family or something and actually have a personal relationship with them because for them this is their life for this period of time and they're confined in this place which can be very dehumanizing... I think I realized that it is so, so, so important.” (Student: Sarah)*

Clinical reasoning skills related to applying the theoretical information in context (i.e. the choice of interventions):

*“...uhm, yeah difficulty prioritizing what is important for that patient at that time. So yeah, individualizing the treatment specific to the patient and their problems, rather than having a general sort of recipe - all ICU patients need this package of care, whatever.” (Wendy, South African clinical expert)*

#### Feeling prepared for ICU clinical work

FG participants expressed feeling overwhelmed by the ICU environment at the start of their clinical rotations, despite having completed the theoretical e-learning module. This was related to specific clinical situations for which they were not prepared, such as wires becoming undetached, alarms going off, or dealing with emergency situations in the ICU.

*“He suddenly ripped out his mechanical ventilation [..] And I didn't know how to put it back in like properly, and where I could touch it, and hold it and oh god that was horrible.” (Student: Leah)*

Clinical experts also experienced undergraduate students feeling anxious and at times, being overly cautious when handling connections to equipment:

*“..in terms of tubes and lines, they're scared to handle [laughs]. I mean yeah [..] they probably err on the side of caution and don't handle as much as they should.” (Wendy, South African clinical expert)*

*“...possibly to pay too much attention to the equipment. So, if, alarms are going off, looking at the equipment rather than looking at the patient [..] They are very awkward handling the equipment, now they do improve, it's a learning curve.” (Amy, Irish clinical expert)*

In the clinical environment, students discovered the reality of the theoretical content covered in the course:

*“First thing I noticed was how common delirium is [..] I was reading about it in the course but I'm like okay, that's a weird thing that* might *happen, but no [..] it was a daily thing.” (Student: Sarah)*

*“When I actually got there and I saw the machines I was like, oh, this is this [..] but then the physios were like okay can you now unhook this person and I was like [..] No! You know, the module did not really prepare you for the handling of the wires.” (Student: Leah)*

Overall, students perceived the e-learning module’s general content on the ICU environment to be helpful in preparation for their clinical rotation. It decreased anxiety:

*“Yes, I felt less scared, I guess. At the beginning I was like I have no idea [..] all those tubes and things and you have no idea how the patients are like, but I feel less scared doing it now [after course completion].” (Student: Diane)*

The e-learning module enabled them to recognize and interpret reporting methods and patient objective data:

*“I was able to see the PT's SOAP notes [..] it was a different layout than I was used to, but the abbreviations and stuff it was exactly the same. I felt prepared for that.” (Student: Sarah)*

*“Actually, for me, I found many assessment tools from the ICU course in my internship site, so with the RASS and stuff they would use it, so that was actually good to hear about it before.” (Student: Jasmine)*

*“I thought the vital signs [..] it was really, really good because [..] especially I saw that in acute care as well, they get on that a lot [..] they were always the things that they were asking me about like: ‘Okay, what do those numbers mean? Why is that important?’ and that was really good to have in the module.” (Student: Ella)*

The course also enabled the students to anticipate the ICU patients’ often complicated recovery process:

*“We had quite a few long-term patients as well so that was actually really useful to learn about all those syndromes. To put it in to perspective what they might get afterwards [..]”. (Student: Olivia)*

#### Dealing with local variety

Dealing with the local variety was a theme identified from the data and related to situations where the general character of the e-learning module did not match with the clinical practice in the variety of international ICU settings. This was illustrated by situations where students were expected to perform clinical tasks, such as respiratory care:

*“I noticed that, and I think it is different from country to country. [..] like suction is sometimes the work of the physio and sometimes the work of the nurse.” (Student: Sarah)*

*“I saw that physios in England were really involved in the weaning process off of the ventilator, a lot more.” (Student: Ella)*

*“[..] I don’t know if respiratory is addressed elsewhere, if not might be worthwhile considering [..] based on our settings in South Africa, sort of 80% of the treatments in the ICU have a respiratory component [..]” (Wendy, South African clinical expert)*

Variety in the use of assessment tools in clinical practice, compared to those covered in the e-learning module, was also noted:

*“in a hospital I was in, they did like a [Manchester] mobility score. [..] they all knew, all physios knew what it meant [..] that's how they scored [..] and then wrote SOAP notes as well, so you could see how the patient was progressing by these numbers.” (Student: Ella)*

*“and I know that [in the Netherlands they] like the DEMMI, but [..] I’m not sure if it would be worth looking at, or if that’s standard practice in sort of other ICUs [..] so if you're working elsewhere [..] the CPAx is quite a nice tool [..] holistic also.” (Wendy, South African clinical expert)*

*“...they don't know about assessing patients, how to assess MRC or uhm hand grip and uhm functional tests and all that stuff.” (Alexandra, Greek clinical expert)*

Participants sometimes experienced discrepancy between the evidence-based content of the e-learning module compared to daily clinical practice:

*“...so we have a competency checklist for respiratory and we also have an ‘on call’ checklist so I use those to kind of guide me. But they’re quite respiratory-oriented. [..] research is getting less and less supportive of our [..] respiratory interventions and more supportive of our rehab interventions.” (Amy, Irish clinical expert)*

*“Germany is not really famous for using evidence-based assessment tools [*laughs*] so maybe... I don't know what they did.” (Student: Leah)*

*“..and it’s a problem that we have in [our country] that [..] sort of the general ICU cultures are quite different, so if you are teaching students now sort of more evidence-based practice in terms of rehabilitation [..], there's quite a lot of resistance [..] from more senior physios and from nursing staff which hinders them [the students] for being able to practice what they [..] have been taught.” (Wendy, South African clinical expert)*

## Discussion

This study confirms that an e-learning module is a feasible and valuable teaching method to prepare international undergraduates for intensive care unit physiotherapy (ICU-PT), as learning objectives with regards to recognition, interpretation, understanding and simple application were achieved. Participants to this study perceived the e-learning module to be helpful in anticipating the ICU environment, patient conditions, and basic PT assessment and intervention tasks. Participants felt less prepared for dealing with emergency situations, handling patients’ lines and attachments, adapting to the variety in clinical expectations across international ICU settings and utilizing higher-level clinical reasoning skills, such as designing tailor-made interventions based on a patient’s clinical presentation. Results from this study align with existing evidence suggesting that supervised practice in clinical settings is required to increase students’ confidence and improve clinical reasoning skills [19, 22, 33, 34]. Integration of this e-learning module with clinical practice provides a foundation for the highly demanding clinical responsibilities in ICU. Timing in which the educational tool is offered in relation to the clinical experience is essential [18, 25]. If, additional to this e-learning module, more complex and clinical reasoning-provoking material would be completed *during* the clinical rotation, learning on the two highest levels of Miller’s pyramid of clinical competence - the ‘shows how’ and the ‘does’ - could likely be facilitated [35].

This study also showed the variety of clinical practice requirements in ICU settings worldwide which provides a challenge for undergraduate curricula in the context of (international) mobility of health professionals [36]. Recent studies highlight these differences; ICU-PTs working in Australia or some European countries are expected to adjust ventilator settings, perform (endo) tracheal suctioning and interpret imaging findings, whereas South African ICU-PTs must also show cultural sensitivity and be a team player [13, 15, 16]. To accommodate for this international variety in clinical practice, e-learning modules prove to be efficient teaching tools, as the content is easily adaptable and additional, elective chapters could be added [25, 28]. For faculties, where research, education and clinical practice are tightly linked, e-learning modules can serve as excellent tools to incorporate the results of ongoing communication between these departments, hence providing students with authentic content in preparation for clinical practice.

### Limitations to this study

The small study sample could be a limitation to generalizability of the results to the student population of undergraduate PT programs worldwide. Experts included in this study also comprised a small sample and the results are therefore not meant to be representative of clinical practice in ICUs and hospitals across the globe. Our study sample consisted of mainly females (93%), which is a slight overrepresentation compared to undergraduate PT students worldwide, although a majority of applicants to PT programs, as well as registered physiotherapists, are of female gender [37–39].

The chosen topics for the expert interviews were purposefully focused towards required student competencies, and this could have led to bias in the results. However, data collection and analysis did not show reasons for inclusion of new topics or themes and therefore confirmed suitability of the initial topic list.

We did not evaluate clinical performance of the students quantitatively and therefore we cannot quantify improvements in cognitive, behavioral or technical (skills) performance measures.

Although the procedures were monitored carefully, selection bias and observer bias cannot be excluded due to the fact that final year students of our undergraduate program were involved in recruitment of participants as well as data collection through focus groups, which consisted of fellow students.

## Conclusion

An evidence-based e-learning module on physiotherapy in the ICU is a feasible and valuable contribution to the undergraduate bachelor program and succeeds in preparing students for their clinical rotation in ICU. The flexibility of this teaching method allows for regular updates to the content and catering for a variety of PT tasks in ICUs worldwide. However, the course did not fully succeed in removing student anxiety when handling complex patient cases; this objective is difficult to achieve with e-learning only. Future endeavors should investigate the feasibility of a closer integration of this e-learning module and clinical practice as well as incorporating additional module chapters to facilitate complex clinical reasoning and clinical performance.

## Data Availability

Data sharing is not applicable to this article as no datasets were generated or analyzed during the current study.

## Abbreviations

ICU: Intensive Care Unit
PT: Physiotherapy / Physical Therapy
ICU-PT: ICU physiotherapist
ESP: European School of Physiotherapy
AUAS: Amsterdam University of Applied Sciences
Amsterdam UMC: Amsterdam University Medical Centers
ICTO: Institute Information Communication Technology and Education
BSc: Bachelor of Science
FG: Focus Group
NVMO-ERB: Netherlands Association for Medical Education Ethical Review Board
EI: Expert Interview
SOAP: acronym for Subjective Objective Assessment Plan (notation method)
RASS: Richmond Agitation and Sedation Scale
DEMMI: DE Morton Mobility Index
CPax: Chelsea critical care Physical Assessment tool
MRC: Medical Research Council
